# The Accessory Gene *saeP* of the SaeR/S Two-Component Gene Regulatory System Impacts *Staphylococcus aureus* Virulence During Neutrophil Interaction

**DOI:** 10.3389/fmicb.2020.00561

**Published:** 2020-04-22

**Authors:** Madison M. Collins, Ranjan K. Behera, Kyler B. Pallister, Tyler J. Evans, Owen Burroughs, Caralyn Flack, Fermin E. Guerra, Willis Pullman, Brock Cone, Jennifer G. Dankoff, Tyler K. Nygaard, Shaun R. Brinsmade, Jovanka M. Voyich

**Affiliations:** ^1^Department of Microbiology and Immunology, Montana State University, Bozeman, MT, United States; ^2^Department of Biology, Georgetown University, Washington, DC, United States

**Keywords:** *sae*, *Staphylococcus aureus*, neutrophil, gene regulation, virulence

## Abstract

*Staphylococcus aureus* (*S. aureus*) causes a range of diseases ranging from superficial skin and soft-tissue infections to invasive and life-threatening conditions (Klevens et al., 2007; Kobayashi et al., 2015). *S. aureus* utilizes the Sae sensory system to adapt to neutrophil challenge. Although the roles of the SaeR response regulator and its cognate sensor kinase SaeS have been demonstrated to be critical for surviving neutrophil interaction and for causing infection, the roles for the accessory proteins SaeP and SaeQ remain incompletely defined. To characterize the functional role of these proteins during innate immune interaction, we generated isogenic deletion mutants lacking these accessory genes in USA300 (USA300Δ*saeP* and USA300Δ*saeQ*). *S. aureus* survival was increased following phagocytosis of USA300Δ*saeP* compared to USA300 by neutrophils. Additionally, secreted extracellular proteins produced by USA300Δ*saeP* cells caused significantly more plasma membrane damage to human neutrophils than extracellular proteins produced by USA300 cells. Deletion of *saeQ* resulted in a similar phenotype, but effects did not reach significance during neutrophil interaction. The enhanced cytotoxicity of USA300Δ*saeP* cells toward human neutrophils correlated with an increased expression of bi-component leukocidins known to target these immune cells. A *saeP* and *saeQ* double mutant (USA300Δ*saePQ*) showed a significant increase in survival following neutrophil phagocytosis that was comparable to the USA300Δ*saeP* single mutant and increased the virulence of USA300 during murine bacteremia. These data provide evidence that SaeP modulates the Sae-mediated response of *S. aureus* against human neutrophils and suggest that *saeP* and *saeQ* together impact pathogenesis *in vivo*.

## Introduction

*Staphylococcus aureus* (*S. aureus*) is a highly-adaptable pathogen able to infect various tissues. Common manifestations of *S. aureus* infections range from mild skin and soft-tissue infections to invasive disease. Additionally, this pathogen has gained resistance against many anti-microbial drugs, leaving healthcare providers with few options for the treatment of infections (Chambers, 2001; Chambers and Deleo, 2009). In the past, antimicrobial-resistant *S. aureus* infections were mostly associated with a recent hospital stay, but the rise in community-associated infections has steadily increased since the late 1990s (Chambers, 2001; King et al., 2006). Drug-resistance combined with the lack of understanding of protective immunity to *S. aureus* has delayed the development of new therapeutics to treat this ubiquitous opportunistic pathogen.

The human neutrophil is essential for resolution of *S. aureus* infections, as individuals suffering from defects in neutrophil function are more susceptible to *S. aureus* infection (Lekstrom-Himes and Gallin, 2000). *S. aureus* has evolved many mechanisms to circumvent killing by these potent innate immune cells. The production of secreted virulence factors during pathogenesis is primarily controlled by the combined influence of two-component systems (TCSs) that sense the host environment and respond accordingly. Of these, the Sae TCS has been shown to be essential for evasion of human neutrophil killing (Voyich et al., 2009; Guerra et al., 2016). SaeR/S is immediately up-regulated following neutrophil phagocytosis and the histidine kinase, SaeS, is thought to specifically recognize neutrophil components (Voyich et al., 2005, 2009; Geiger et al., 2008; Mainiero et al., 2010; Cho et al., 2015; Zurek et al., 2015). The response regulator, SaeR, is activated following phosphorylation by SaeS and subsequently alters gene transcription by directly binding to a specific recognition sequence in the promoter region of numerous virulence genes including *nuc* and the bi-component leukotoxins *lukF* (PVL), *lukGH* (*lukAB*), and *hlgBC* that target human neutrophils (Nygaard et al., 2010; Olson et al., 2013; Liu et al., 2016). The upregulation of these genes facilitates *S. aureus* survival following neutrophil phagocytosis (Voyich et al., 2009; Flack et al., 2014). However, the *sae* locus also includes two accessory genes, *saeP* and *saeQ*, whose gene products are not entirely understood. Previously published *in vitro* studies suggest these proteins form a complex with SaeS that deactivates SaeR (Jeong et al., 2012). It has also been shown that increased expression of *saeP* impacts biofilm formation by increasing retention of high molecular weight DNA on the biofilm surface (Kavanaugh et al., 2019). The same study also demonstrated increases in *saeP* gene expression correlated with decreases in nuclease activity during biofilm development (Kavanaugh et al., 2019). Considering the importance of Sae during neutrophil interactions, we investigated the importance of *saeP* and *saeQ* during challenge with human neutrophils and *in vivo* using murine models of invasive disease and skin and soft-tissue infection. For these studies, we used *S. aureus* strain LAC, a USA300 isolate, as USA300 is the dominant clone causing community-associated methicillin resistant *S. aureus* (CA-MRSA) disease in the United States (David and Daum, 2010). Results demonstrate that deletion of *saeP* increased *S. aureus* cytotoxicity against neutrophils *ex vivo*. Moreover, the deletion of both *saeP* and *saeQ* markedly increased both nuclease expression in kidneys and overall mortality following intravenous infection.

## Results

### Generation of USA300 Isogenic Mutants Deficient in Either *saeP* or *saeQ*

**FIGURE 1:**
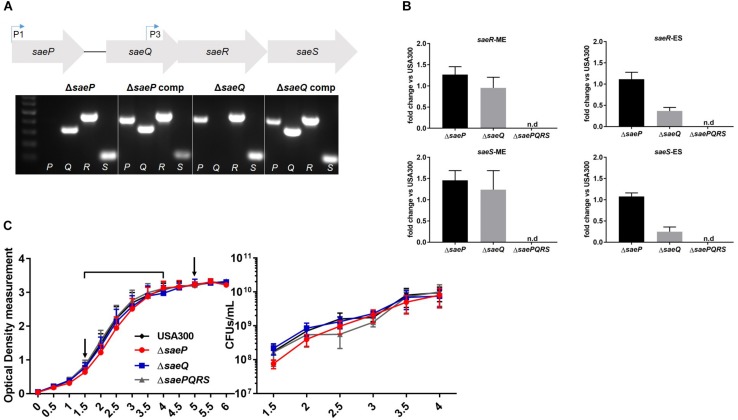
Generation of an isogenic *saeQ* mutant. **(A)** (Above) Schematic of the four genes of the *sae* locus. Bent arrows indicate transcriptional start sites described in Novick and Jiang (2003), Steinhuber et al. (2003), and Jeong et al. (2011). (Below) Results of agarose gel electrophoresis showing USA300 mutants lacking *saeP* (from Kavanaugh et al., 2019), *saeQ*, and respective complemented strains. Letters at the bottom of the gel indicate the *sae* gene targeted by PCR analysis. Primer sequences are included in Table 1. **(B)** TaqMan® RT-PCR analysis of *saeS* and *saeR* transcript levels in USA300Δ*saeP* and USA300Δ*saeQ* relative to USA300 at mid-exponential phase (ME) and early stationary phase (ES). Transcript levels in samples were analyzed in triplicate and results are from two independent experiments. **(C)** *In vitro* growth of USA300, USA300Δ*saeP*, USA300Δ*saeQ*, and USA300Δ*saePQRS* (Flack et al., 2014) measured by optical density at 600 nm (OD_600_) (left) and colony forming units (CFUs) (right). Timepoints are indicated by the bracket on the OD_600_ plot. Arrows indicate time points for RNA harvest (used in **B**). Data are presented as the mean ± SEM of seven independent experiments. n.d., not detected.

The Sae TCS is composed of four genes: *saeP, saeQ, saeR*, and *saeS* (Figure 1A). Although *saeS* and *saeR*, encoding the sensory kinase and response regulator respectively, have been demonstrated to be essential in *S. aureus* virulence and pathogenesis (Voyich et al., 2009; Nygaard et al., 2010, 2018; Flack et al., 2014; Liu et al., 2015, 2016; Zurek et al., 2015; Guerra et al., 2016), surprisingly little research has been performed on the two accessory genes of the Sae system, *saeP* and *saeQ*. Published data indicate that *saeP* encodes a lipoprotein anchored to the exterior surface of the plasma membrane, whereas *saeQ* encodes a transmembrane protein (Jeong et al., 2012; Kavanaugh et al., 2019). To investigate the roles of these genes, we utilized a *saeP* deletion mutant (USA300Δ*saeP*) generated previously in Kavanaugh et al. (2019), and deleted the first third of *saeQ* (164 bp) in USA300 LAC using allelic replacement to create an isogenic *saeQ* deletion mutant (USA300Δ*saeQ*) that preserves the P3 promoter (Bae and Schneewind, 2006; Jeong et al., 2011). Absence of *saeP* and *saeQ* genes were verified by PCR (Figure 1A). Importantly, deletion of *saeP* or *saeQ* did not substantially impact *saeR* and *saeS* gene expression. TaqMan® real-time RT-PCR analyses indicated a slight increase in *saeR* transcript levels in the USA300Δ*saeP* mutant at both mid-exponential (ME) and early stationary (ES) phases of growth relative to USA300. This trend was also established for the expression of *saeS* in USA300Δ*saeP* at ME phase. Expression of *saeR* and *saeS* were essentially unchanged in USA300Δ*saeQ* relative to USA300 at ME but were decreased during ES (Figure 1B). Basal expression from the P3 promoter is likely unaffected by deletion of *saeQ* since the deletion ends 108 base pairs upstream of the P3 promoter (Jeong et al., 2011) and ME expression is similar to USA300. When the Sae TCS is activated, transcription from the P1 promoter increases (Novick and Jiang, 2003; Steinhuber et al., 2003). Therefore, when the system activates in ES (Flack et al., 2014), mRNA transcripts containing the *saeQ* deletion may have reduced stability since the mRNA secondary structure has recently been shown to be important (Marincola and Wolz, 2017). Nevertheless, this is not expected to affect the activation of SaeR/S target genes, as overexpression of *saeRS* does not alter the expression profile of the Sae-regulon (Mainiero et al., 2010; Liu et al., 2016). Additionally, USA300Δ*saeP* and USA300Δ*saeQ* strains showed no significant growth defects compared to USA300 (Figure 1C).

**TABLE 1:** Primers used to generate *S. aureus* mutant strains, respective complemented strains, and TaqMan® primer and probe sequences.

**Primer**	**Sequence**	**Description**	**References**
**Construction of USA300Δ*saeP* and USA300Δ*saeQ***			
Forward	5′-GTTGTTGAATTCACCTGATACATTACAGACC-3′	600 bp upstream of *saeP*	Kavanaugh et al., 2019
Reverse	5′-CAGAAATTGAGTACTAGATCTGTATTCATGCTAACTCCTCATTTC-3′	Upstream of *saeP* plus overlap	Kavanaugh et al., 2019
Forward	5′-GAATACAGATCTAGTACTCAATTTCTGAGTTAAACTTTTATTTACAAC-3′	Downstream of *saeP* plus overlap	Flack, 2014
Reverse	5′-GTTGTTGGTACCAAGAAACTAGCAGCATATGC-3′	600 bp downstream of *saeP*	Flack, 2014
Forward	5′-GTTGTTGAATTCCCTAACAGGTACATTCAGTTC-3′	EcoR1, 600 bp upstream of *saeQ*	Flack, 2014
Reverse	5′-GCGAGTACTAGATCTCATTCTTTCTATTATTGTGTGTAATTTATAT-3′	Upstream of *saeQ* plus overlap	Flack, 2014
Forward	5′-AGAATGAGATCTAGTACTCGCAAATATAGTTGCACATAC-3′	165 bp into *saeQ* plus overlap	Flack, 2014
Reverse	5′-GTTGTTGGTACCGATGGTATATGTTGTAAAGCTCTC-3′	*Kpn*I, 900 bp downstream of *saeQ*	This work
Forward	5′-TAATTTAGCGCCGCCGAAGA-3′	*saeP*	This work
Reverse	5′-TTTTTAGCAGCTGGTGCTGT-3′	*saeP*	This work
Forward	5′- CTCTGTTCTTACGACCTCTAAAGTAAT-3′	*saeQ*	This work
Reverse	5′-GTTTAGTACCAGTCATCGCTAACA-3′	*saeQ*	This work
Forward	5′-GGTGGTGAATTCTTAACTTATCAAATTGAAGAAATGAGGAGTTAGC-3′	pEPSA5-*saeP*-EcoR1	This work
Reverse	5′-ACCACCGGATCCAATTGATTATTTTAATTTAGCGCCGCC-3′	pEPSA5-*saeP*-*Bam*HI	This work
Forward	5′-GGTGGTGAATTCTTATATAAATTACACACAATAAATAGAAAGAATGTGAACATC-3′	pEPSA5-*saeQ*-EcoR1	This work
Reverse	5′-GGTGGTGGATCCTGTTCATCATCCACGATCAGTAAGT-3′	pEPSA5-*saeQ*-*Bam*HI	This work
**Taqman® primer/probe sequences**			
Forward	5′-CACCTAACAGGTACATTCAGTTCTA-3′	*saeP* primer	This work
Reverse	5′-GGTAGACGTATAAATCTGGACCTTT-3′	*saeP* primer	This work
Probe	5′-ACGGTGAAACTGTTGAAGGTAAAGCTGA-3′	*saeP* probe	This work
Forward	5′-CACCAGAGTGGTATAAGTGGTT-3′	*saeQ* primer	This work
Reverse	5′-CAAAGCCTCCAAAGAAACTAGC-3′	*saeQ* primer	This work
Probe	5′-TTGTTGTCCCACTCGGAGAGATTGC-3′	*saeQ* probe	This work

### Deletion of *saeP* Increases Bacterial Survival Following Neutrophil Phagocytosis

**FIGURE 2:**
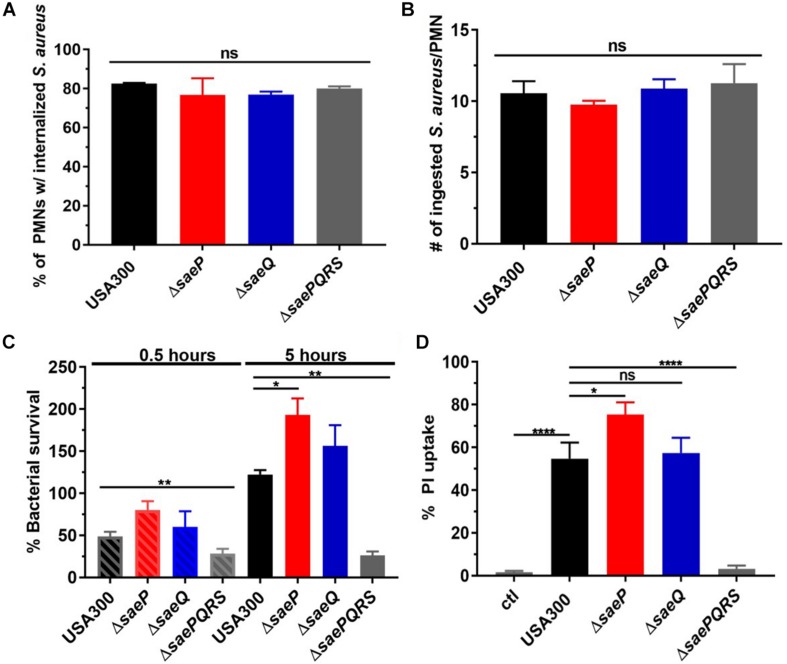
Deletion of *saeP* significantly increases *S. aureus* survival and toxicity during neutrophil interaction. **(A)** Percent *S. aureus* ingested by neutrophils. Samples were collected on an Image Stream® Imaging Flow Cytometer and *S. aureus* internalization was analyzed using the IDEAS software® as described in Materials and Methods for three independent experiments. **(B)** Numbers of ingested *S. aureus* per PMN are shown. The average number of *S. aureus* detected to be ingested per PMN is ∼ 10 for all strains. Data are presented as the mean ± SEM of three independent experiments. **(C)** Percent survival for the indicated strains and timepoints is shown. Bacterial survival is significantly increased in USA300Δ*saeP* compared to USA300 at 5 h following phagocytosis. Survival was calculated with the following equation: (CFU + PMN at time_n_/CFU + PMN at time_0_) × 100 (as in Voyich et al., 2005). Data are presented as the mean ± SEM of six independent experiments. At 0.5 h, USA300Δ*saePQRS* was significantly different from USA300 **P ≤ 0.001 using one-way ANOVA with Tukey’s post-test. At 5 h, *P ≤ 0.05 and **P ≤ 0.001 relative to USA300 using one-way ANOVA and Tukey’s post-test. **(D)** Supernatants from USA300Δ*saeP* cultures cause significantly more neutrophil plasma membrane damage compared to supernatants from USA300 cultures. *S. aureus* strains were grown to early stationary phase, supernatants harvested as described in Materials and Methods (diluted 1:10), and incubated with neutrophils for 1 h. Propidium iodide (PI) uptake was assessed by flow cytometry. Data are presented as the mean ± SEM of five independent experiments. *P ≤ 0.05, ****P ≤ 0.0001 using one-way ANOVA with Tukey’s post-test. ctl, neutrophil-only control. ns, not significant.

SaeS has been shown to be activated by neutrophil phagocytosis and associated components including alpha-defensin and hydrogen peroxide (Voyich et al., 2005; Geiger et al., 2008; Zurek et al., 2014). Moreover, deletion of *saeR/S* has been shown to significantly decrease *S. aureus* survival and cytolytic capacity following neutrophil phagocytosis (Voyich et al., 2009; Flack et al., 2014). However, nothing is known about how SaeP and SaeQ contribute to staphylococcal neutrophil evasion. To determine the role of these accessory proteins during interaction with human neutrophils, we initially evaluated phagocytosis and killing of USA300Δ*saeP*, USA300Δ*saeQ*, USA300Δ*saePQRS* (Flack et al., 2014), or USA300. Importantly, there were no significant differences in the uptake of these strains by human neutrophils (Figures 2A,B), consistent with previous observations using Δ*saeR/S* strains that have shown the SaeR/S system has no significant impact on neutrophil phagocytosis (Voyich et al., 2009; Guerra et al., 2016). Next, we assessed *S. aureus* survival after neutrophil phagocytosis. After 30 min, we measured very modest increases in the survival of the *saeP* and *saeQ* mutant strains relative to the parental wild-type strain. However, deletion of *saeP* significantly increased bacterial survival compared to USA300 5-h after phagocytosis. There was also a noticeable although not statistically significant increase in survival of USA300Δ*saeQ* compared to USA300 at 5 h post-neutrophil exposure. We measured a significant reduction in survival of USA300Δ*saePQRS* strain at both timepoints, confirming the importance of *saeR/S* for *S. aureus* survival following neutrophil phagocytosis as previously observed (Voyich et al., 2009; Figure 2C).

### Deletion of *saeP* Increases Production of Neutrophil Cytolytic Factors

SaeR/S up-regulates the transcription of numerous secreted virulence factors including the bi-component leukotoxins LukG/H, PVL, and HlgB/C that specifically target and disrupt the neutrophil plasma membrane (Voyich et al., 2009; Nygaard et al., 2010; Sun et al., 2010; Ventura et al., 2010; Flack et al., 2014; Zurek et al., 2014). Since USA300Δ*saeP* cells demonstrated increased survival following phagocytosis, we hypothesized that *saeP* might influence the production of secreted cytolytic factors (i.e., ability to permeabilize neutrophils). Indeed, neutrophils exposed to filtered supernatants taken from ES cultures of the USA300Δ*saeP* mutant exhibited significantly more plasma membrane damage than neutrophils exposed to supernatants taken from cultures of USA300 as determined by propidium iodide uptake (Figure 2D). Supernatants from USA300Δ*saeQ* showed no significant differences in cytolytic activity compared with USA300. Confirming previous observations with Δ*saeR/S* strains, neutrophils exposed to the supernatants from USA300Δ*saePQRS* cultures showed significantly reduced plasma membrane damage compared to results from exposure to culture supernatants from all other *S. aureus* strains tested and similar to that of neutrophils not exposed to *S. aureus* supernatants (Voyich et al., 2009; Flack et al., 2014). USA300Δ*saeP* complemented with *saeP* in trans reduced the cytotoxicity of this strain to levels that paralleled USA300 (Supplementary Figure S1A).

### Deletion of *saeP* Increases Transcript Abundance of Several Leukotoxins

**FIGURE 3:**
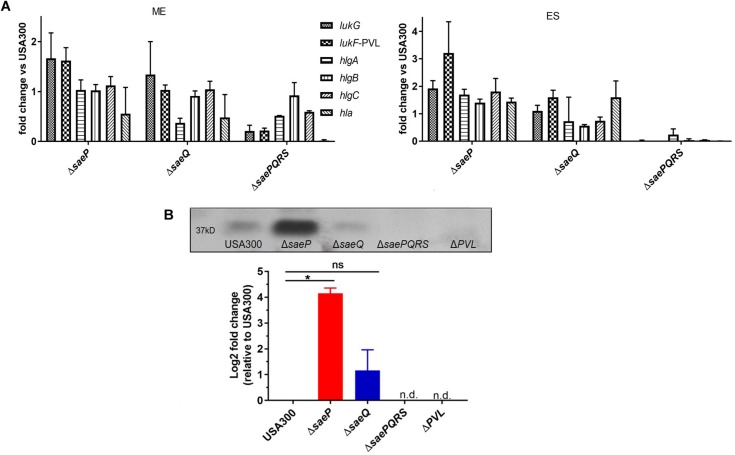
USA300Δ*saeP* demonstrates increased transcript abundance of several known SaeR/S-virulence factors. **(A)** Mean fold-change of known SaeR/S-regulated virulence genes in USA300Δ*saeP* and USA300Δ*saeQ* relative to USA300 is shown. Transcript abundance was measured using TaqMan® RT-PCR at mid-exponential (ME; left) and early stationary (ES; right) phases of *in vitro* growth. Transcripts were normalized to *gyrB* and calibrated to transcript abundance in USA300. Data are from at least two independent experiments. **(B)** The USA300Δ*saeP* strain of *S. aureus* produces significantly more Panton Valentine Leukocidin (PVL) compared to USA300. (Top) Representative Western blot of PVL protein in supernatants from *S. aureus* grown overnight. (Bottom) Quantification of PVL was calculated using densitometry as described in Materials and Methods. Data shown are amounts relative to USA300 and presented as the mean ± SEM of three independent experiments. *P ≤ 0.05 One-Way ANOVA with Tukey’s post-test. n.d., not detected.

The SaeR/S system is essential for transcriptional regulation of virulence factors known to impact neutrophil function (Voyich et al., 2009; Mainiero et al., 2010; Nygaard et al., 2010; Flack et al., 2014; Zurek et al., 2014; Guerra et al., 2016). Since the USA300Δ*saeP* strain demonstrated increased survival following phagocytosis by neutrophils, and supernatants from USA300Δ*saeP* had increased cytolytic activity toward neutrophils, we profiled the transcript abundance of select SaeR/S-regulated virulence factors known to impact neutrophil viability. During mid-exponential phase (ME) we measured subtle increases in *lukG* transcript abundance in both USA300Δ*saeP* and USA300Δ*saeQ* mutant strains, as well as a subtle increase in *lukF*-PVL transcript abundance levels in USA300Δ*saeP* (Figure 3A). Importantly, we measured more pronounced increases in *lukG*, *lukF-PV*, *hlgA*, *hlgB*, and *hlg*C transcript abundance in USA300Δ*saeP* relative to USA300 during early stationary (ES) phase. Transcript abundance of all select virulence genes was reduced in the USA300Δ*saePQRS* mutant in accordance with previous observations examining the influence of Sae on virulence gene transcription (Voyich et al., 2009; Nygaard et al., 2010; Flack et al., 2014; Zurek et al., 2014). Expression of *saeP* in trans in the USA300Δ*saeP* strain reduced transcript abundance and cytotoxicity to levels at or below those measured in USA300 (Supplementary Figures S1B,C).

Supporting transcript analysis of *lukF-PV*, secreted PVL in overnight culture supernatants was significantly increased in the USA300Δ*saeP* strain compared to USA300 (Figure 3B). As anticipated, PVL was essentially undetectable in culture supernatants from the Δ*saePQRS* mutant, demonstrating a strong dependency on Sae for production of PVL (Figure 3B).

### Deletion of *saeQ* Attenuates Mortality in Mice Following Intravenous Infection

**FIGURE 4:**
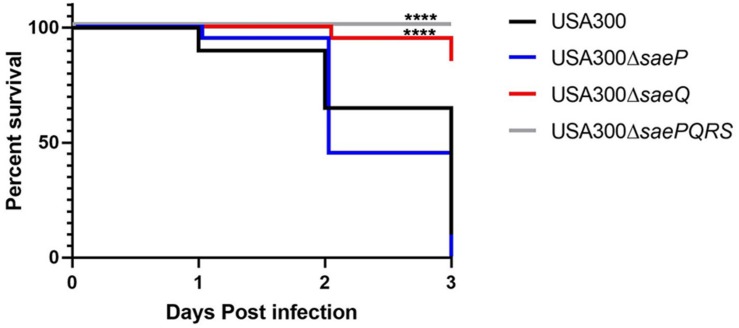
*saeQ* contributes to staphylococcal disease during bacteremia. Groups of 10 C57BL/6 mice were infected intravenously via the tail vein with 1 × 10^7^ CFU of the indicated *S. aureus* strains. Survival curves are from two independent experiments ****P < 0.0001, log-rank (Mantel-Cox) test.

To investigate the individual roles of *saeP* and *saeQ* during staphylococcal disease, we used a well-established model of acute bacteremia (Voyich et al., 2009; Nygaard et al., 2010). Mice (groups of 10) were infected intravenously with 1 × 10^7^ CFUs of either *S. aureus* USA300, USA300Δ*saeP*, USA300Δ*saeQ*, or USA300 Δ*saePQRS*. Consistent with previous studies (Nygaard et al., 2010), ∼65% of the mice infected with USA300 died within 48 h, and on average fewer than 10% of the mice survived 72 h post-infection. Although there were no significant differences in the mortality of mice challenged with USA300Δ*saeP* compared with USA300, nearly all mice infected with USA300Δ*saeQ* survived 72 h post-infection (Figure 4). All mice challenged with Δ*saePQRS* survived, congruent with previous studies (Voyich et al., 2009; Nygaard et al., 2010) and demonstrating the critical role of the Sae system following bloodstream infection (Figure 4).

SaeR/S is also critical for *S. aureus* pathogenesis during murine skin and soft-tissue infection (SSTI) (Voyich et al., 2009; Nygaard et al., 2010, 2018). To investigate the importance of SaeP and SaeQ in SSTI, BALB/c and C57BL/6 mice were infected subcutaneously with 1 × 10^7^ CFUs of either USA300 or our isogenic mutants, and abscess area was monitored for 10 days. While we measured a significant decrease in the abscess area of mice infected with the USA300Δ*saePQRS* mutant compared to USA300, we detected no significant differences in abscess size or incidence of dermonecrosis when either *saeP* or *saeQ* were deleted (Supplementary Figures S2A,B). Taken together, although Sae TCS activity is required for full virulence in both bacteremia and SSTI, our data indicate SaeP and SaeQ are dispensable during SSTI, but SaeQ appears to be important during bacteremia.

### USA300Δ*saePQ* Mimics the USA300Δ*saeP* Phenotype During Neutrophil Interaction, but Significantly Increases Mortality Following Intravenous Infection in Mice

**FIGURE 5:**
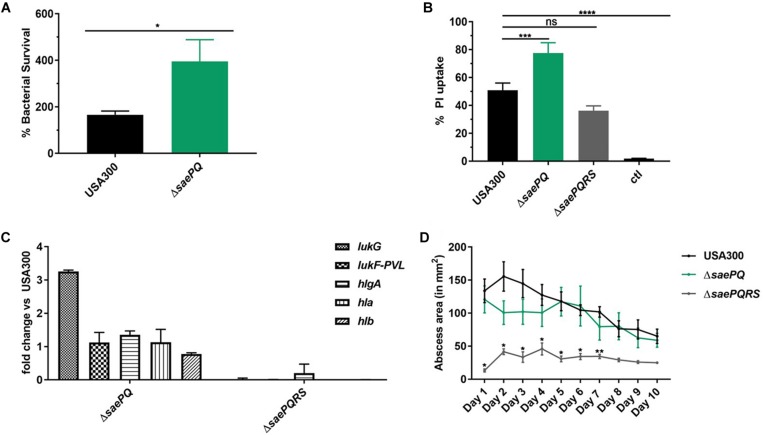
USA300Δ*saePQ* demonstrates enhanced survival following neutrophil phagocytosis. **(A)** USA300Δ*saePQ* survives neutrophil killing significantly better than USA300 after 5 h incubation. Data are presented as the mean ± SEM of eight independent experiments. **(B)** PMN plasma membrane damage was significantly increased in neutrophils infected with USA300Δ*saePQ* compared to infection with USA300 (neutrophil: bacteria ratio of 1:5). Propidium iodide uptake was assessed at 3 h post-infection by flow cytometry and indicate USA300Δ*saePQ* produces significantly more cytolytic proteins in the supernatants than USA300. Data are presented as the mean ± SEM of five independent experiments. **(C)** Transcript abundance of *lukG* is increased in USA300Δ*saePQ* relative to expression in USA300 at early stationary phase. Gene transcripts were normalized to *gyrB* and calibrated to the expression levels of USA300. **(D)** Deletion of *saePQ* did not significantly impact abscess area. C57BL/6 mice were infected subcutaneously with 1 × 10^7^ CFU of *S. aureus*. Abscess area was measured daily and results shown are the average of five mice per group. *P ≤ 0.05, **P ≤ 0.01, ***P ≤ 0.001, ****P ≤ 0.0001 via one-way ANOVA Tukey’s post-test. Data are presented as the mean ± SEM.

**FIGURE 6:**
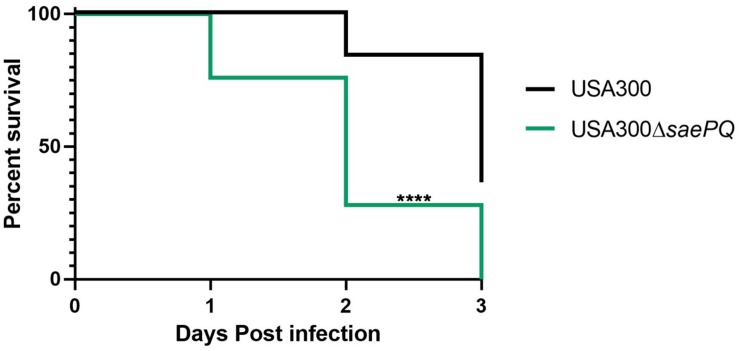
Deletion of *saePQ* enhances the virulence in *S. aureus* in a mouse model of systemic infection. Survival curves for C57BL/6 mice (*n* = 13/group) challenged with either 1 × 10^7^ CFU *S. aureus* or USA300 or USA300Δ*saePQ* via tail vein injection. Data shown are death from infection and euthanasia (for exceeding scores on the welfare rubric) during the 72 h endpoint. Data are from two independent experiments. ****P < 0.0001, log-rank (Mantel-Cox) test.

Due to our observations that USA300Δ*saeP* demonstrates increased ability to survive neutrophil phagocytosis and that USA300Δ*saeQ* had increased (although not significant) survival following neutrophil phagocytosis, we wondered whether a double mutant deficient in both *saeP* and *saeQ* might exhibit an enhanced phenotype in the aforementioned neutrophil assays. To test this, we first deleted the entire *sae* locus in LAC using allelic exchange and then introduced *saeRS* driven by their native P3 promoter into the chromosome at the *geh* locus using the single copy integration plasmid pCL55 (Liu et al., 2015). The resulting strain (hereafter referred to as USA300 Δ*saePQ*) exhibited reduced expression of *saeR/S* during exponential growth *in vitro* (Supplementary Figures S3A,B). This could be due to the absence of transcriptional readthrough from the stronger P1 promoter. Regardless, the USA300 Δ*saePQ* double mutant is still capable of inducing SaeR/S-mediated virulence gene expression in response to human neutrophil peptide-1 (HNP-1) exposure (Supplementary Figure S3C). Compared to USA300, USA300 Δ*saePQ* exhibited a significant increase in both bacterial survival following phagocytosis as well as increased cytotoxicity toward neutrophils. This increase in virulence was consistent with observations made with USA300Δ*saeP* in both neutrophil survival and plasma membrane damage (Figure 5 compared to Figure 2). We measured similar fold-changes in the expression of Sae-dependent virulence genes in USA300Δ*saePQ* (also similar to those observed in USA300Δ*saeP*) during ES compared to USA300 (compare *lukG* and *lukF-PVL* in Figure 5 and Figure 2). As we saw in the SSTI model following challenge with the USA300Δ*saeP* or USA300Δ*saeQ* mutants, mice challenged with the USA300Δ*saePQ* mutant showed no significant differences in abscess area compared to USA300 (Figure 5D). However, we found that intravenous infection with the USA300Δ*saePQ* double mutant led to a significant increase in mortality compared to infection with USA300 in the bacteremia model (Figure 6).

To investigate if the increased mortality in mice challenged with the USA300Δ*saePQ* mutant was due to increased expression of SaeR/S-dependent virulence factors, we infected mice with either USA300 or USA300Δ*saePQ* mutant carrying an integrated *nuc-gfp* translational fusion (Behera et al., 2019). Three days post-infection, groups of three-eight C57BL/6 mice were euthanized. Kidneys were harvested, fixed and embedded, and examined using confocal microscopy. Consistent with previous results, USA300 formed clearly-defined abscesses containing a nidus of staphylococci also referred to as a staphylococcal abscess community (SAC). These bacteria exhibit spatial regulation of *nuc-gfp* as we previously reported (Behera et al., 2019). That is, we detected strong expression of *nuc-gfp* in the core of the SAC and muted expression on the periphery of the SAC. On the other hand, the USA300Δ*saePQ* mutant failed to form discrete, well-formed abscesses. Instead, widespread infiltration of the renal tissues was apparent, and the *nuc-gfp* reporter was highly expressed in bacteria on the periphery of the lesions (Supplementary Figure S4). Taken together, these data indicate that the *saePQ* mutant phenocopies a *saeP* mutant during human neutrophil interaction, and lack of both gene products impacts pathogenesis and virulence expression during bacteremia.

## Discussion

The Sae TCS of *S. aureus* contributes to the expression and production of virulence and immunomodulatory factors that are essential for *S. aureus* neutrophil evasion and pathogenesis (Voyich et al., 2009; Nygaard et al., 2010; Sun et al., 2010; Flack et al., 2014; Zurek et al., 2014). Much is known about the molecular genetics of activation of the SaeS bifunctional kinase/phosphatase and the SaeR response regulator. However, despite its discovery over two decades ago, relatively little is known about how the auxiliary proteins SaeP and SaeQ contribute to Sae TCS activity and staphylococcal disease. Herein, we utilized *saeP* and *saeQ* single and double mutant strains to characterize the role of these accessory proteins using both *ex vivo* human neutrophil assays and *in vivo* mouse models of infection. Our results suggest that SaeP acts as a regulator of SaeR/S-dependent virulence during challenge with human neutrophils. Although no statistically significant phenotype could be established for USA300Δ*saeQ* following interactions with human neutrophils, we did note an increase in the survival of mice following intravenous infection with this strain, suggesting that SaeQ contributes to SaeR/S signaling during bacteremia.

Our findings support previously-published *in vitro* data (Jeong et al., 2012; Kavanaugh et al., 2019) suggesting SaeP may regulate SaeR/S-dependent effectors during human neutrophil encounters. The observation that the USA300Δ*saeP* strain is more cytotoxic against neutrophils and exhibits increased bacterial survival following phagocytosis suggest that the impact of SaeP on SaeR-target genes is specific and likely dependent on environmental cues. The observation that there was no additive effect of enhanced survival following neutrophil phagocytosis of USA300Δ*saePQ* compared to USA300Δ*saeP* also supports a specific role for *saeP* during interaction with human neutrophils. Future studies will continue to characterize the role of SaeQ.

*S. aureus* uses secreted nuclease (Nuc) along with secreted adenosine synthase (AdsA) to escape neutrophil extracellular traps (NETs) (Thammavongsa et al., 2013). Increasing Nuc expression and production in kidney tissues may increase the production of deoxyadenosine, and this could trigger caspase-3-mediated immune cell death (Thammavongsa et al., 2013). In USA300 LAC, SaeP down-regulated *nuc-gfp* gene expression via SaeR/S (Kavanaugh et al., 2019). Thus, the hypervirulent phenotype of the *saePQ* double mutant during acute bacteremia is not unexpected. However, it is unclear why the *saeP* and *saeQ* single mutants behave differently. Since we used a purified diet for the *in vivo nuc-gfp* mouse reporter studies, we cannot exclude the possibility that this diet amplifies mild phenotypes observed in mice challenged with the *saeP* and *saeQ* mutant strains. It would be interesting to examine the impact of SaeP and SaeQ during chronic kidney infections in mice. The observation that the *saeQ* mutant is highly attenuated was unexpected. Given the known protein-protein interactions between SaeQ and SaeS, it is possible that the stability and/or activity of SaeS is compromised in this mutant. Indeed, SaeQ is required for hyperactive SaeS (SaeS^L18P^) stability in strain Newman (Jeong et al., 2011, 2012). Clearly there is still much to learn about the function of these proteins; only a few studies investigate SaeP and/or SaeQ.

Recently, Kavanaugh et al. (2019) confirmed the cellular localization of SaeP on the cell surface as a lipoprotein, and that its C-terminal domain is facing the extracellular matrix. SaeQ is predicted to be a membrane protein with three membrane-spanning domains, and forms a complex with SaeP and SaeS in the membrane (Jeong et al., 2012). A conserved domain search revealed that the C-terminal portion of SaeP looks like a member of the DM13 superfamily of proteins. Because of its association with the DOMON domain, is thought DM13 proteins might be involved in electron transfer. SaeQ is predicted to be a member of the DoxX family of proteins similar to *Bacillus subtilis* putative oxidoreductases MhqP and CatD, and *Escherichia coli* inner membrane proteins YphA and YqjF (Iyer et al., 2007). The Sae system is responsive to cellular respiratory status but the mechanism is unclear. One model posits that inhibition of respiration by oxygen depletion or chemical disruption of the electron transport chain by reactive oxygen species or nitrosative species could lead to a block in the respiratory chain and a buildup of reduced quinones in the membrane, activating Sae activity (Mashruwala et al., 2017). It is conceivable SaePQ sense this perturbation, go inactive, and promote SaeS kinase activity. Alternatively, SaeP possesses a pI of ∼8 and is capable of binding negatively charged eDNA in acidic environments (Kavanaugh et al., 2019). It is tempting to speculate that a physical interaction with neutrophil NET DNA induces some conformational change in SaeP that hinders its ability to stimulate phosphatase activity of SaeS in the staphylococci nearest the neutrophil cuff. Either repressive mechanism could explain the apparent increased *nuc-gfp* expression in the periphery of lesions formed by the *saePQ* double mutant (Supplementary Figure S4, compare panels D vs. A). Increased nuclease expression may result in increased virulence during immune cell encounter and could explain why no discrete abscesses could be found when mice were infected with the *saePQ* double mutant (Supplementary Figure 4).

*In vivo* observations made with USA300Δ*saePQ* suggest that neither *saeP* nor *saeQ* influence virulence factors that contribute to murine skin and soft-tissue abscess severity. Inasmuch as alpha-toxin (Hla) is known to play a key role in dermonecrosis caused by USA300 during murine skin and soft tissue infection (Kennedy et al., 2010), results from the skin infection model are in agreement with our gene expression data that demonstrate *sae*P and *sae*Q do not influence *hla* transcript abundance (Supplementary Figures S2A,B). However, the observation that USA300Δ*saePQ* is hypervirulent in the bacteremia model is more difficult to explain with our current data. Potentially, different host niches have varying levels of different activating cues and levels of Sae TCS activity. Clearly, additional studies are needed to precisely determine the importance and impact of SaeP and SaeQ at these sites *in vivo* and to identify conditions that influence their expression and function.

## Materials and Methods

### Bacterial Strains and Culture

All *S. aureus* strains used in this study are derivatives of the clinically-relevant CA-MRSA strain USA300 (LAC) that was previously cured of the plasmid encoding erythromycin resistance (Boles et al., 2010). Unless otherwise indicated, overnight and subculture media consisted of tryptic soy broth (TSB) (EMD Millipore; Darmstadt, Germany) supplemented with 0.5% (w/v) glucose. When needed, antibiotics were included in the medium at the following concentrations: ampicillin (Amp), 50 μg ml^–1^; chloramphenicol (Cm), 5 μg ml^–1^; and erythromycin (Erm), 5 μg ml^–1^. Subcultures were created using 1:100 dilution of the overnight culture. For the growth curves, OD_600_ readings were collected every 0.5 h using a Nanodrop 2000C UV-Vis Spectrophotometer (ThermoFisher Scientific; Wilmington, DE, United States) or an Amersham Ultraspec 2100 pro UV-visible spectrophotometer and colony forming units (CFUs) were enumerated after incubation overnight at 37°C with 5% CO_2_ as described (Voyich et al., 2005).

### Generation of Mutant Strains

Construction of the isogenic *saeQ* deletion mutant was performed using allelic exchange and pJB38 plasmid (Bae and Schneewind, 2006; Bose et al., 2013). The *saeP* mutant was constructed previously (Kavanaugh et al., 2019). To construct the *saePQ* strain, we first deleted the entire *sae* operon using pKOR1-sae (Bae and Schneewind, 2006). Next, we transduced the strain to chloramphenicol resistance, moving in the P_saeP__3_-*saeRS* construct (*saeRS* under the control of their native promoter, cloned into pCL55 and integrated into the *geh* locus, Cm^R^). Then, we integrated the *nuc-gfp* reporter as described by allelic exchange (Behera et al., 2019). Briefly, DNA fragments upstream and downstream of the gene or gene fragment of interest were amplified using primers listed in Table 1, purified by agarose gel electrophoresis, then combined in a two-step overlap PCR reaction and cloned into pJB38 (Flack et al., 2014). Δ*saePQRS* was made previously in Flack et al. (2014). The resulting plasmid was transformed sequentially into *Escherichia coli* (*E. coli*) strain ER2566 (New England Biolabs), then *S. aureus* strain RN4220, and the final background USA300 LAC (Flack, 2014). Final mutants were verified by PCR amplification of the chromosomal region of interest and DNA sequencing. Lack of *saeP* and *saeQ* in the mutant strains were verified by PCR and agarose gel electrophoresis.

For complementation strains, the *saeP* and *saeQ* genes were cloned into the pEPSA5 plasmid (Forsyth et al., 2002) using restriction enzymes (EcoR1 and BamH1) and primers listed in Table 1. The resulting plasmids (pEPSA5-*saeP*comp and pEPSA5-*sae*Qcomp) drive expression of the *sae* genes from the xylose-inducible P_xyl_ promoter. To induce expression, the medium was supplemented with 2% (w/v) xylose in experiments involving these strains as indicated (Forsyth et al., 2002). These plasmids were transformed into electrocompetent *E. coli* GM2163 (New England Biolabs), then directly into the respective mutant *S. aureus* strain (USA300Δ*saeP* and USA300Δ*saeQ*) via *E. coli* strain IM08B (Monk and Foster, 2012) and called pEPSA5-*saeP*comp and pEPSA5-*saeQ*comp. The resulting strains were confirmed using PCR amplification and agarose gel electrophoresis, and presence of transcript abundance verified by TaqMan RT-PCR as done previously (Voyich et al., 2009; Nygaard et al., 2010; Flack et al., 2014).

### Neutrophil Isolation

Heparinized venous blood from healthy donors was collected in accordance with a protocol approved by the Institutional Review Board for Human Subjects at Montana State University. All donors provided written consent to participate in the study. Human neutrophils [polymorphonuclear leukocytes (PMNs)] were isolated under endotoxin-free conditions (<25pg ml^–1^) as previously described (Voyich et al., 2005, 2009). Purity (<1% PBMC contamination) and viability (<2% propidium iodide positivity) of neutrophil preparations were assessed by flow cytometry on a FACS Calibur instrument and BD Biosciences Cell Quest Pro software (version 0.3.3f1b).

### Image Stream Phagocytosis Assay

Neutrophil phagocytosis was determined using a fluorescence-based flow cytometry/microscopy method described previously (Ploppa et al., 2011). Briefly, *S. aureus* was grown to mid-exponential phase, opsonized with 50% (vol/vol) normal human serum and labeled with 750 μL fluorescein isothiocyanate (FITC) at a final concentration of 0.002 mg mL^–1^. *S. aureus* strains were combined with neutrophils at a multiplicity of infection (MOI) of 10:1 (bacteria: neutrophils) in 96-well plates coated with human serum coated (20% v/v). Phagocytosis was synchronized by centrifugation as described (Voyich et al., 2005) and incubated at 37°C with 5% CO_2_ for 30 min. Cells were fixed in 2% (v/v) Periodate-Lysine-Paraformaldehyde (PLP) for 10 min at room temperature (Pieri et al., 2002). PLP was then washed away and antibodies/stains were applied: mouse anti-human CD11b antibody-PE (BD; Franklin Lakes, NJ, United States) and nuclear stain DRAQ5^TM^ (ThermoFisher Scientific; Wilmington, DE, United States). Cells were washed and suspended in 50 μL sterile Dulbecco’s phosphate buffered saline (DPBS) and analyzed by an ImageStream®X Mark II Imaging Flow Cytometer (Millipore Sigma) the following day. Phagocytosis was analyzed using IDEAS® software (AMNIS®, Millipore Sigma, Darmstadt, Germany) where cell images were gated to include neutrophils that were both in focus and singlets. Of these cells, images fluorescing both neutrophil and *S. aureus* membrane dyes were analyzed using the AMNIS internalization wizard (Ploppa et al., 2011).

### Bacterial Survival Assay

Bacterial survival was assessed following synchronized phagocytosis as previously described (Voyich et al., 2005). Briefly, *S. aureus* strains were grown to mid-exponential phase, opsonized in 50% (v/v) normal human serum, and combined with neutrophils in 96-well plates coated with 20% (v/v) human serum (MOI of 10:1) and incubated at 37°C with 5% CO_2_. At indicated times, 11 μL of 2% (w/v) saponin solution was added to each well and incubated for 15 min on ice. Samples were sheared using a 1 mL syringe with a blunt needle and bacteria were enumerated by dilution on tryptic soy agar (TSA) following overnight incubation at 37°C with 5% CO_2_.

### Plasma Membrane Damage

Propidium iodide (PI) uptake was used as a measure of plasma membrane permeability to assess damage of neutrophils by secreted *S. aureus* proteins as described (Nygaard et al., 2012, 2018; Flack et al., 2014). Briefly, bacterial strains were cultured at 37°C for 5 h with shaking (250 RPM) in TSB. After, 1 × 10^9^ CFUs of bacteria were collected and centrifuged for 5 min at 8,000 × g. Supernatants were sterile-filtered and diluted (as indicated in figure legends) with DPBS and exposed to neutrophils for 1 h at 37°C with 5% CO_2_. After incubation, cells were stained with 0.5 μL PI (1 mg mL^–1^ Life Technologies) and analyzed by flow cytometry on a FACS Calibur flow cytometer (BD Biosciences; Franklin Lakes, New Jersey). Neutrophil membrane damage was also assessed by flow cytometry using whole bacteria. For these experiments, neutrophils were exposed to live bacteria (MOI 5:1) and incubated for 3 h at 37°C with 5% CO_2_.

### Transcriptional Analysis of Target Genes

TaqMan® gene expression experiments were performed as previously described (Voyich et al., 2005, 2009; Nygaard et al., 2010). Relative quantification of *S. aureus* target genes was determined by the change in expression of target transcripts normalized to that of the housekeeping gene [gyrase B (*gyrB*)] and relative to USA300 LAC transcript levels. Primer/probe sequences are described in Table 1 (Voyich et al., 2009; Nygaard et al., 2010). Where indicated, transcript abundance was also measured using SYBR Green chemistry and the absolute transcript abundance method as indicated and as described in Mlynek et al. (2018).

### Western Blot Analysis

Supernatants from overnight cultures in TSB without supplemented glucose were harvested as described above, total protein was measured (Pierce BCA Protein Assay) and adjusted to 500 μg mL^–1^. Samples (14 μL) were resolved using 12% SDS-PAGE gels, (100 V for 45 min) and transferred onto nitrocellulose (at 10 mAmps overnight). Membranes were washed and blocked in DPBS containing 5% (w/v) milk solution for 1 h followed by incubation with rabbit anti-LukS-PV primary antibody (abcam; Cambridge, MA, United States) at a concentration of 0.6 μg mL^–1^ (4 h at 4°C). PVL was detected after 1 h incubation with goat anti-rabbit IgG coupled to horseradish peroxidase (at 1:10,000 dilution) (Jackson ImmunoResearch; West Grove, PA, United States) and developed using 5 mL 3,3′,5,5′-tetramethylbenzidine (TMB) substrate. Images were taken with an Gel Doc Imager (ProteinSimple; San Jose, CA, United States) and analyzed by ImageJ densitometry software (Schindelin et al., 2012).

### GFP Reporter Assays With HNP-1

Bacteria were grown to exponential phase (OD_600_ ∼ 0.6–0.8) as described previously (Waters et al., 2016) in 250 ml DeLong flasks containing dilute Luria broth (Geiger et al., 2008) (5:1 flask:medium ratio) with vigorous shaking (280 RPM) at 37°C in a water bath. Cultures were diluted to a starting OD_600_ ∼ 0.1 in fresh medium and aliquoted into individual wells of a 96 well plate (cultures of 200 μl each) and incubated in a computer controlled Tecan F200 plate reader at 37°C. The optical density at 600 nm (OD_600_) and GFP fluorescence (485 nm excitation, 535 nm emission) values were read every 15 min after shaking (15 s, 2 mm amplitude). When OD_600_ values reached ∼0.4, the plate was removed, and the indicated wells were spiked with 5 μg ml^–1^ of the human neutrophil peptide-1 (HNP-1) or vehicle and returned to the plate reader. Data acquisition continued for an additional 12 h. Mean ± SEM relative fluorescence units (RFUs; GFP fluorescence/OD_600_) from three independent experiments are reported.

### Mouse Infection Models

#### Skin and Soft-Tissue Infection (SSTI)

All studies conformed to NIH guidelines and were approved by the Institutional Animal Care and Use Committee at Montana State University. Female C57BL/6 mice (12 weeks old) were purchased from Charles River Laboratories and maintained at the Animal Resources Center at Montana State University. Female and male BALB/C mice were purchased from the Animal Resources Center at Montana State University. *S. aureus* strains: USA300 LAC, and isogenic mutants USA300Δ*saeP*, USA300Δ*saeQ*, USA300Δ*saePQRS*, USA300Δ*saePQ* strains were grown to mid-exponential phase, washed twice with sterile DPBS and resuspended in DPBS at a concentration of 1 × 10^7^ cells per 50 μL. The dose was confirmed by plating serial dilutions on TSA plates For the abscess model, mice (groups of five) were shaved and inoculated with *S. aureus* subcutaneously into the lower back (Voyich et al., 2009; Nygaard et al., 2010). Infected area was measured using the formula: (l × w).

#### Bacteremia Model

Experiments were performed following a protocol approved by the Animal Care and Use Committee at Georgetown University (GUACUC). *S. aureus* strains were grown to exponential phase in 250 ml DeLong shake flasks (5:1 flask: medium ratio), harvested at OD_600_ ∼0.4–0.6, washed twice in sterile phosphate buffer saline (PBS), and resuspended to an appropriate optical density equivalent to 1 × 10^8^ colony forming units (CFUs) ml^–1^. Groups of female C57BL/6 mice (6–8-weeks old, purchased from Charles River Laboratories) were infected intravenously via the tail vein with ∼1 × 10^7^ cells in 100 μl of sterile PBS. The dose was confirmed by plating serial dilutions on TSA plates. Animals were monitored twice daily and evaluated following a GUACUC-approved scoring rubric. Infections were allowed to progress for 72 h or until humane endpoints were reached.

To analyze *nuc-gfp* expression in tissues, infections were performed as described for USA300 LAC and the Δ*saePQ* double mutant essentially described above (and specifically in Behera et al., 2019); notably, animals were fed AIN-93 purified diet (Reeves et al., 1993). Briefly, mice were euthanized 72 h post-infection and kidneys were harvested, fixed with 10% (v/v) buffered formalin, embedded in Sub Xero clear tissue freezing medium (Mercedes Medical), and sectioned into 10 μm slices. Sections were mounted with 4′,6-diamidino-2-phenylindole (DAPI) stain and imaged using laser confocal scanning microscopy. Images were processed using ImageJ (Schindelin et al., 2012). Excitation wavelengths for the fluorescence channels are as follows: DAPI, 405 nm; GFP, 488 nm. Emitted fluorescence data were collected over the following ranges of wavelengths: DAPI, 419–481 nm, GFP, 505–551 nm.

### Statistics

Statistical analyses were performed using GraphPad Prism version 8.0 (GraphPad Software, La Jolla, CA, United States) with *t*-tests and ANOVA as indicated. Error bars represent the standard error of the mean (SEM).

## Data Availability Statement

The datasets generated for this study are available on request to the corresponding authors.

## Ethics Statement

The studies involving human participants were reviewed and approved by Institutional Review Board for Human Subjects at Montana State University. The patients/participants provided their written informed consent to participate in this study.

## Author Contributions

MC, JV, RB, and SB contributed to the conception and design of this study. MC, RB, TE, OB, BC, WP, CF, KP, FG, TN, and JD performed the experiments and data analysis. MC, JV, and SB wrote and prepared the manuscript for submission. All authors read and approved this manuscript.

## Conflict of Interest

The authors declare that the research was conducted in the absence of any commercial or financial relationships that could be construed as a potential conflict of interest.

## Supplementary Material

FIGURE S1 Complementation of *saeP* and *saeQ* restores USA300 phenotype. Gene complementation with *saeP* and *saeQ* on the xylose-inducible pEPSA5 plasmid restores USA300 phenotype. Promoter expression induced with 2% (w/v) xylose in the medium. **(A)** Gene expression of *saeP* and *saeQ* is rescued in *S. aureus* mutant strains complemented with *saeP or saeQ* expressed in trans. **(B)** Complementation of *saeP* restores transcript of select *S. aureus* genes to levels observed in USA300 (or higher). Transcript abundance is relative to *gyrB* and calibrated to expression in USA300 (*n* = 2/gene). **(C)** Complementation of *saeP* and *saeQ* in trans reduces secreted cytolytic factors that target neutrophil plasma membrane damage to levels secreted by USA300. Data are presented as the mean ± SEM of four independent experiments. Stats: One-Way ANOVA with Tukey’s post-test; ns, not significant.

FIGURE S2 Deletion of *saeP* or *saeQ* did not significantly impact abscess area. Deletion of *saeP* or *saeQ* did not significantly impact abscess area. **(A)** BALB/C mice (5 per group) and **(B)** C57BL/6 mice (5 per group) were infected subcutaneously with 1 × 10^7^ CFUs of each *S. aureus* strain and abscess area was subsequently monitored for 10 days. Abscess area was measured daily and results shown are the average area per strain. Representative images are from C57BL/6 mice on day 2. Graphs represent data from two biological replicates for BALB/c and one for C57BL/6. Unpaired *t*-test relative to USA300, ^∗^*p*-value ≤ 0.05, ^∗∗^*p*-value ≤ 0.01. Data are presented as the mean ± SEM.

FIGURE S3 The Δ*saePQ* mutant has reduced *saeR/S* transcript levels but induces Sae-dependent genes normally. **(A,B)** Normalized transcript abundance is shown for USA300 and the indicated mutant strains of *S. aureus* grown to exponential phase using SYBR green qRT-PCR as described in Materials and Methods and as described in Mlynek et al. (2018). Data indicate the mean ± SEM from three independent experiments. Statistical analysis: ANOVA (ordinary one-way), Dunnett’s multiple comparison test, asterisks indicate the level of significance compared to the USA300 (*P* ≤ 0.05). For USA300Δ*saePQRS*, the transcript level is below the limit of detection. **(C)** The indicated strains carrying a *nuc-gfp* reporter fusion were grown to exponential phase (OD_600_ ∼ 0.4) in dilute Luria broth, at which time bacteria were exposed to either vehicle (water) or HNP-1 for 12 h. The data shown are the mean relative fluorescence units (RFUs; fluorescence/OD_600_) ±SEM for three independent experiments performed in technical triplicate; statistical significance was assessed by paired *t*-test (^∗^*P* ≤ 0.05, ^∗∗^*P* ≤ 0.01, ^∗∗∗^*P* ≤ 0.001). ND, not detected: signal was below the limit of detection.

FIGURE S4 Deletion of *saePQ* influences lesion structure and *nuc* expression in infected kidneys. **(A–F)** Representative confocal micrographs of staphylococcal lesions in kidney produced by USA300 and USA300Δ*saePQ*. Channels: *nuc-sGFP* **(A,D)**, DAPI **(B,E)**, and merge **(C,F)** (scale bar is 25 μm). The fluorescence image acquisition parameters used are as follows; excitation for DAPI (blue) and GFP (green) are 405 and 488 nm respectively; emission ranges are 419–481 nm (DAPI) and 505–551 nm (GFP).

## References

[B1] Bae T. Schneewind O. (2006). Allelic replacement in *Staphylococcus aureus* with inducible counter-selection. *Plasmid* 55 58–63. 10.1016/j.plasmid.2005.05.005 16051359

[B2] Behera R. K. Mlynek K. D. Linz M. S. Brinsmade S. R. (2019). A fluorescence-based method to study bacterial gene regulation in infected tissues. *J. Vis. Exp.* 144:e59055. 10.3791/59055 30855576 PMC7295204

[B3] Boles B. R. Thoendel M. Roth A. J. Horswill A. R. (2010). Identification of genes involved in polysaccharide-independent *Staphylococcus aureus* biofilm formation. *PLoS One* 5:e10146. 10.1371/journal.pone.0010146 20418950 PMC2854687

[B4] Bose J. L. Fey P. D. Bayles K. W. (2013). Genetic tools to enhance the study of gene function and regulation in *Staphylococcus aureus*. *Appl. Environ. Microbiol.* 79 2218–2224. 10.1128/AEM.00136-13 23354696 PMC3623228

[B5] Chambers H. F. (2001). The changing epidemiology of *Staphylococcus aureus*? *Emerg. Infect. Dis.* 7 178–182. 10.3201/eid0702.010204 11294701 PMC2631711

[B6] Chambers H. F. Deleo F. R. (2009). Waves of resistance: *Staphylococcus aureus* in the antibiotic era. *Nat. Rev. Microbiol.* 7 629–641. 10.1038/nrmicro2200 19680247 PMC2871281

[B7] Cho H. Jeong D. W. Liu Q. Yeo W. S. Vogl T. Skaar E. P. (2015). Calprotectin increases the activity of the SaeRS two component system and murine mortality during *Staphylococcus aureus* infections. *PLoS Pathog.* 11:e1005026. 10.1371/journal.ppat.1005026 26147796 PMC4492782

[B8] David M. Z. Daum R. S. (2010). Community-associated methicillin-resistant *Staphylococcus aureus*: epidemiology and clinical consequences of an emerging epidemic. *Clin. Microbiol. Rev.* 23 616–687. 10.1128/CMR.00081-09 20610826 PMC2901661

[B9] Flack C. E. (2014). *Mutagenesis and structural analysis of the Staphylococcus Aureus Sae two-component system reveals the intricate nature of virulence regulation.* Ph.D. thesis, University of Iowa, Iowa City, IA.

[B10] Flack C. E. Zurek O. W. Meishery D. D. Pallister K. B. Malone C. L. Horswill A. R. (2014). Differential regulation of staphylococcal virulence by the sensor kinase SaeS in response to neutrophil-derived stimuli. *Proc. Natl. Acad. Sci. U.S.A.* 111 E2037–E2045. 10.1073/pnas.1322125111 24782537 PMC4024872

[B11] Forsyth R. A. Haselbeck R. J. Ohlsen K. L. Yamamoto R. T. Xu H. Trawick J. D. (2002). A genome-wide strategy for the identification of essential genes in *Staphylococcus aureus*. *Mol. Microbiol.* 43 1387–1400. 10.1046/j.1365-2958.2002.02832.x 11952893

[B12] Geiger T. Goerke C. Mainiero M. Kraus D. Wolz C. (2008). The virulence regulator Sae of *Staphylococcus aureus*: promoter activities and response to phagocytosis-related signals. *J. Bacteriol.* 190 3419–3428. 10.1128/JB.01927-07 18344360 PMC2395011

[B13] Guerra F. E. Addison C. B. de Jong N. W. M. Azzolino J. Pallister K. B. van Strijp J. A. G. (2016). *Staphylococcus aureus* SaeR/S-regulated factors reduce human neutrophil reactive oxygen species production. *J. Leukoc. Biol.* 100 1–6. 10.1189/jlb.4VMAB0316-100RR 27334228 PMC5069094

[B14] Iyer L. M. Anantharaman V. Aravind L. (2007). The DOMON domains are involved in heme and sugar recognition. *Bioinformatics* 23 2660–2664. 10.1093/bioinformatics/btm411 17878204

[B15] Jeong D. W. Cho H. Jones M. B. Shatzkes K. Sun F. Ji Q. (2012). The auxiliary protein complex SaePQ activates the phosphatase activity of sensor kinase SaeS in the SaeRS two-component system of *Staphylococcus aureus*. *Mol. Microbiol.* 86 331–348. 10.1111/j.1365-2958.2012.08198.x 22882143 PMC3468659

[B16] Jeong D. W. Cho H. Lee H. Li C. Garza J. Fried M. (2011). Identification of the P3 promoter and distinct roles of the two promoters of the SaeRS two-component system in *Staphylococcus aureus*. *J. Bacteriol.* 193 4672–4684. 10.1128/JB.00353-11 21764914 PMC3165640

[B17] Kavanaugh J. S. Flack C. E. Lister J. Ricker E. B. Ibberson C. B. Jenul C. (2019). Identification of extracellular DNA-binding proteins in the biofilm matrix. *mBio* 10:e01137-19. 10.1128/mBio.01137-19 31239382 PMC6593408

[B18] Kennedy A. D. Wardenburg J. B. Gardner D. J. Long D. Whitney A. R. Braughton K. R. (2010). Targeting of Alpha-hemolysin by active or passive immunization decreases severity of USA300 skin infection in a mouse model. *J. Infect. Dis.* 202 1050–1058. 10.1086/656043 20726702 PMC2945289

[B19] King M. D. Humphrey B. J. Wang Y. F. Kourbatova E. V. Ray S. M. Blumberg H. M. (2006). Emergence of community-acquired methicillin-resistant *Staphylococcus aureus* USA 300 clone as the predominant cause of skin and soft-tissue infections. *Ann. Intern. Med.* 144 309–317. 16520471 10.7326/0003-4819-144-5-200603070-00005

[B20] Klevens R. M. Morrison M. A. Nadle J. Petit S. Gershman K. Ray S. (2007). Invasive methicillin-resistant *Staphylococcus aureus* infections in the United States. *JAMA* 298 1763–1771. 10.1001/jama.298.15.1763 17940231

[B21] Kobayashi S. D. Malachowa N. Deleo F. R. (2015). Pathogenesis of *Staphylococcus aureus* abscesses. *Am. J. Pathol.* 185 1518–1527. 10.1016/j.ajpath.2014.11.030 25749135 PMC4450319

[B22] Lekstrom-Himes J. A. Gallin J. I. (2000). Immunodeficiency diseases caused by defects in phagocytes. *N. Engl. J. Med.* 343 1703–1714. 10.1056/NEJM200012073432307 11106721

[B23] Liu Q. Cho H. Yeo W. S. Bae T. (2015). The extracytoplasmic linker peptide of the sensor protein SaeS tunes the kinase activity required for staphylococcal virulence in response to host signals. *PLoS Pathog.* 11:e1004799. 10.1371/journal.ppat.1004799 25849574 PMC4388633

[B24] Liu Q. Yeo W. S. Bae T. (2016). The SaeRS two-component system of *Staphylococcus aureus*. *Genes* 7:E81. 10.3390/genes7100081 27706107 PMC5083920

[B25] Mainiero M. Goerke C. Geiger T. Gonser C. Herbert S. Wolz C. (2010). Differential target gene activation by the *Staphylococcus aureus* two-component system *saeRS*. *J. Bacteriol.* 192 613–623. 10.1128/JB.01242-09 19933357 PMC2812464

[B26] Marincola G. Wolz C. (2017). Downstream element determines RNase y cleavage of the *saePQRS* operon in *Staphylococcus aureus*. *Nucleic Acids Res.* 45 5980–5994. 10.1093/nar/gkx296 28453818 PMC5449607

[B27] Mashruwala A. A. Gries C. M. Scherr T. D. Kielian T. Boyd J. M. (2017). SaeRS is responsive to cellular respiratory status and regulates fermentative biofilm formation in *Staphylococcus aureus*. *Infect. Immun.* 85:e000157-17. 10.1128/IAI.00157-17 28507069 PMC5520442

[B28] Mlynek K. D. Sause W. E. Moormeier D. E. Sadykov M. R. Hill K. R. Torres V. J. (2018). Nutritional regulation of the Sae two-component system by CodY in *Staphylococcus aureus*. *J. Bacteriol.* 200:e00012-18. 10.1128/JB.00012-18 29378891 PMC5869476

[B29] Monk I. R. Foster T. J. (2012). Genetic manipulation of staphylococci-breaking through the barrier. *Front. Cell. Infect. Microbiol.* 2:49. 10.3389/fcimb.2012.00049 22919640 PMC3417578

[B30] Novick R. P. Jiang D. (2003). The staphylococcal *saeRS* system coordinates environmental signals with agr quorum sensing. *Microbiology* 149 2709–2717. 10.1099/mic.0.26575-0 14523104

[B31] Nygaard T. K. Borgogna T. R. Sward E. W. Guerra F. E. Dankoff J. G. Collins M. M. (2018). Aspartic acid residue 51 of SaeR is essential for *Staphylococcus aureus* virulence. *Front. Microbiol.* 9:3085. 10.3389/FMICB.2018.03085 30619166 PMC6302044

[B32] Nygaard T. K. Pallister K. B. DuMont A. L. DeWald M. Watkins R. L. Pallister E. Q. (2012). Alpha-toxin induces programmed cell death of human T cells, B cells, and monocytes during USA300 infection. *PLoS One* 7:e0036532. 10.1371/journal.pone.0036532 22574180 PMC3344897

[B33] Nygaard T. K. Pallister K. B. Ruzevich P. Griffith S. Vuong C. Voyich J. M. (2010). SaeR binds a consensus sequence within virulence gene promoters to advance USA300 pathogenesis. *J. Infect. Dis.* 201 241–254. 10.1086/649570 20001858 PMC2798008

[B34] Olson M. E. Nygaard T. K. Ackermann L. Watkins R. L. Zurek O. W. Pallister K. B. (2013). *Staphylococcus aureus* nuclease is an SaeRS-dependent virulence factor. *Infect. Immun.* 81 1316–1324. 10.1128/IAI.01242-12 23381999 PMC3639593

[B35] Pieri L. Sassoli C. Romagnoli P. Domenici L. (2002). Use of periodate-lysine-paraformaldehyde for the fixation of multiple antigens in human skin biopsies. *Eur. J. Histochem.* 46 365–375. 12597622 10.4081/1749

[B36] Ploppa A. George T. C. Unertl K. E. Nohe B. Durieux M. E. (2011). ImageStream cytometry extends the analysis of phagocytosis and oxidative burst. *Scand. J. Clin. Lab. Invest.* 71 362–369. 10.3109/00365513.2011.572182 21473709

[B37] Reeves P. G. Nielsen F. H. Fahey G. C. (1993). AIN-93 purified diets for laboratory rodents: final report of the American Institute of nutrition Ad Hoc writing committee on the reformulation of the AIN-76A rodent diet. *J. Nutr.* 123 1939–1951. 10.1093/jn/123.11.1939 8229312

[B38] Schindelin J. Arganda-Carreras I. Frise E. Kaynig V. Longair M. Pietzsch T. (2012). Fiji: an open-source platform for biological-image analysis. *Nat. Methods* 9 676–682. 10.1038/nmeth.2019 22743772 PMC3855844

[B39] Steinhuber A. Goerke C. Bayer M. G. Döring G. Wolz C. (2003). Molecular architecture of the regulatory locus sae of *Staphylococcus aureus* and its impact on expression of virulence factors. *J. Bacteriol.* 185 6278–6286. 10.1128/JB.185.21.6278-6286.2003 14563862 PMC219404

[B40] Sun F. Li C. Jeong D. Sohn C. He C. Bae T. (2010). In the *Staphylococcus aureus* two-component system sae, the response regulator SaeR binds to a direct repeat sequence and DNA binding requires phosphorylation by the sensor kinase SaeS. *J. Bacteriol.* 192 2111–2127. 10.1128/JB.01524-09 20172998 PMC2849438

[B41] Thammavongsa V. Missiakas D. M. Schneewind O. (2013). *Staphylococcus aureus* degrades neutrophil extracellular traps to promote immune cell death. *Science* 342 863–866. 10.1126/science.1242255 24233725 PMC4026193

[B42] Ventura C. L. Malachowa N. Hammer C. H. Nardone G. A. Robinson M. A. Kobayashi S. D. (2010). Identification of a novel *Staphylococcus aureus* two-component leukotoxin using cell surface proteomics. *PLoS One* 5:e11634. 10.1371/journal.pone.0011634 20661294 PMC2905442

[B43] Voyich J. M. Braughton K. R. Sturdevant D. E. Whitney A. R. Saïd-Salim B. Porcella S. F. (2005). Insights into mechanisms used by *Staphylococcus aureus* to avoid destruction by human neutrophils. *J. Immunol.* 175 3907–3919. 10.4049/jimmunol.175.6.3907 16148137

[B44] Voyich J. M. Vuong C. DeWald M. Nygaard T. K. Kocianova S. Griffith S. (2009). The SaeR/S gene regulatory system is essential for innate immune evasion by *Staphylococcus aureus*. *J. Infect. Dis.* 199 1698–1706. 10.1086/598967 19374556 PMC2799113

[B45] Waters N. R. Samuels D. J. Behera R. K. Livny J. Rhee K. Y. Sadykov M. R. (2016). A spectrum of CodY activities drives metabolic reorganization and virulence gene expression in *Staphylococcus aureus*. *Mol. Microbiol.* 101 495–514. 10.1111/mmi.13404 27116338 PMC5007081

[B46] Zurek O. W. Nygaard T. K. Watkins R. L. Pallister K. B. Torres V. J. Horswill A. R. (2014). The role of innate immunity in promoting SaeR/S-mediated virulence in i. *J. Innate Immun.* 6 21–30. 10.1159/000351200 23816635 PMC4435966

[B47] Zurek O. W. Pallister K. B. Voyich J. M. (2015). *Staphylococcus aureus* inhibits neutrophil-derived IL-8 to promote cell death. *J. Infect. Dis.* 212 934–938. 10.1093/infdis/jiv124 25722299 PMC4548456

