# Supplementary figures and images for: The Accessory Gene saeP of the SaeR/S Two-Component Gene Regulatory System Impacts Staphylococcus aureus Virulence During Neutrophil Interaction
Source: Front Microbiol. 2020 Apr 22;11:561. doi: 10.3389/fmicb.2020.00561 (PMC7189620; doi:10.3389/fmicb.2020.00561)

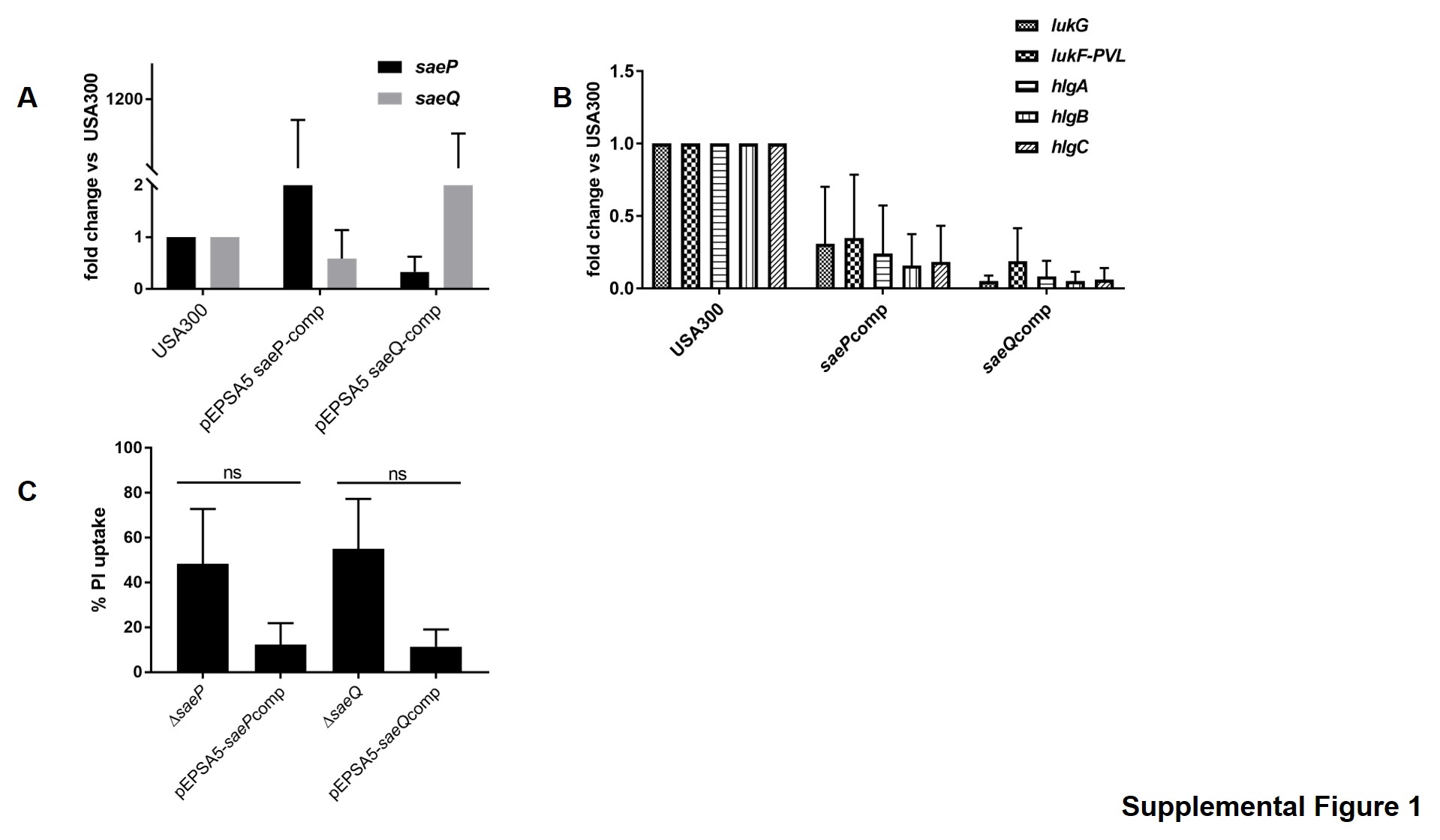

Supplement: FIGURE S1 — Complementation of saeP and saeQ restores USA300 phenotype. Gene complementation with saeP and saeQ on the xylose-inducible pEPSA5 plasmid restores USA300 phenotype. Promoter expression induced with 2% (w/v) xylose in the medium. (A) Gene expression of saeP and saeQ is rescued in S. aureus mutant strains complemented with saeP or saeQ expressed in trans. (B) Complementation of saeP restores transcript of select S. aureus genes to levels observed in USA300 (or higher). Transcript abundance is relative to gyrB and calibrated to expression in USA300 (n = 2/gene). (C) Complementation of saeP and saeQ in trans reduces secreted cytolytic factors that target neutrophil plasma membrane damage to levels secreted by USA300. Data are presented as the mean ± SEM of four independent experiments. Stats: One-Way ANOVA with Tukey’s post-test; ns, not significant. [file Image_1.jpeg]

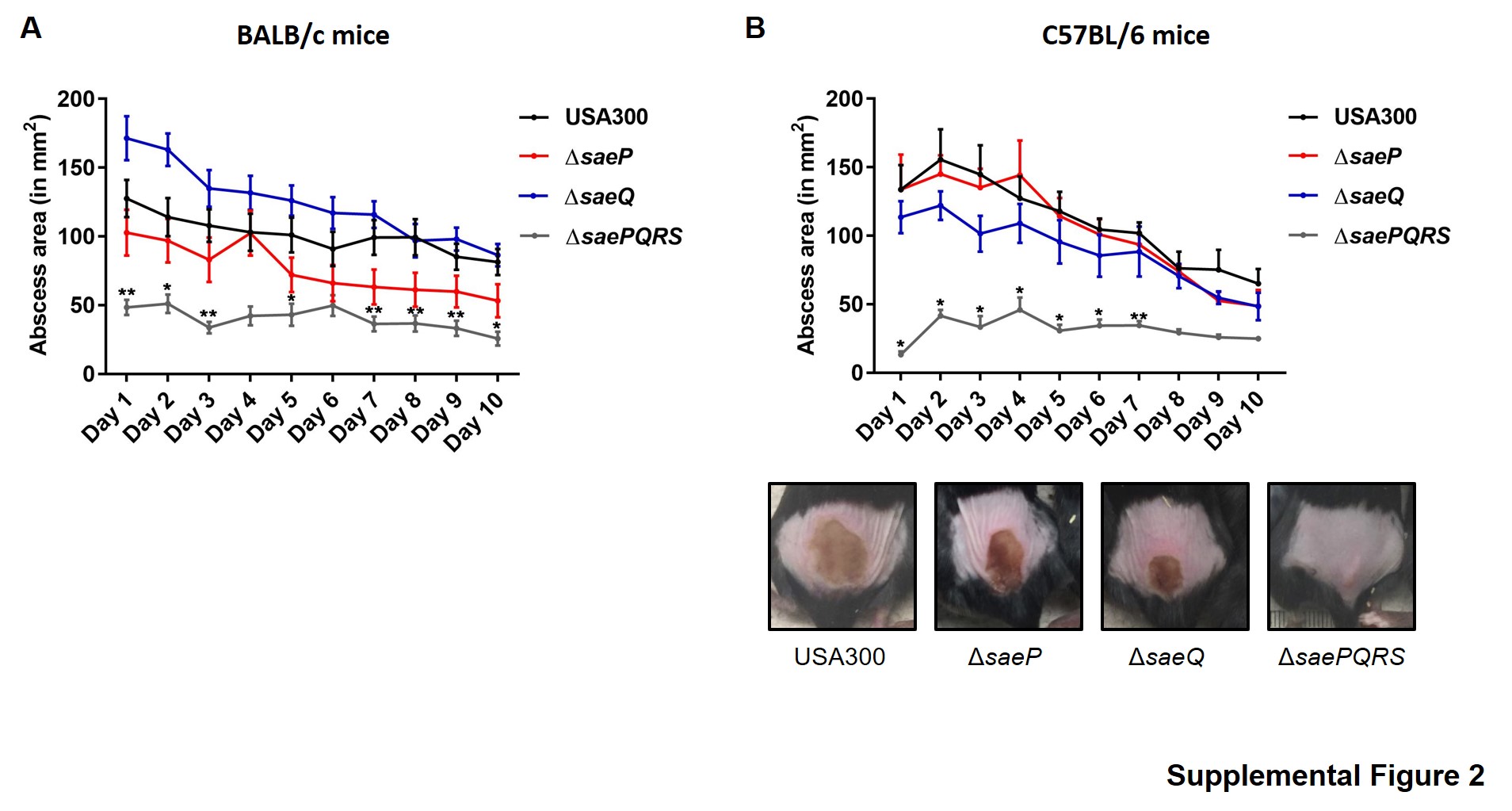

Supplement: FIGURE S2 — Deletion of saeP or saeQ did not significantly impact abscess area. Deletion of saeP or saeQ did not significantly impact abscess area. (A) BALB/C mice (5 per group) and (B) C57BL/6 mice (5 per group) were infected subcutaneously with 1 × 10^7^ CFUs of each S. aureus strain and abscess area was subsequently monitored for 10 days. Abscess area was measured daily and results shown are the average area per strain. Representative images are from C57BL/6 mice on day 2. Graphs represent data from two biological replicates for BALB/c and one for C57BL/6. Unpaired t-test relative to USA300, ∗p-value ≤ 0.05, ∗∗p-value ≤ 0.01. Data are presented as the mean ± SEM. [file Image_2.jpeg]

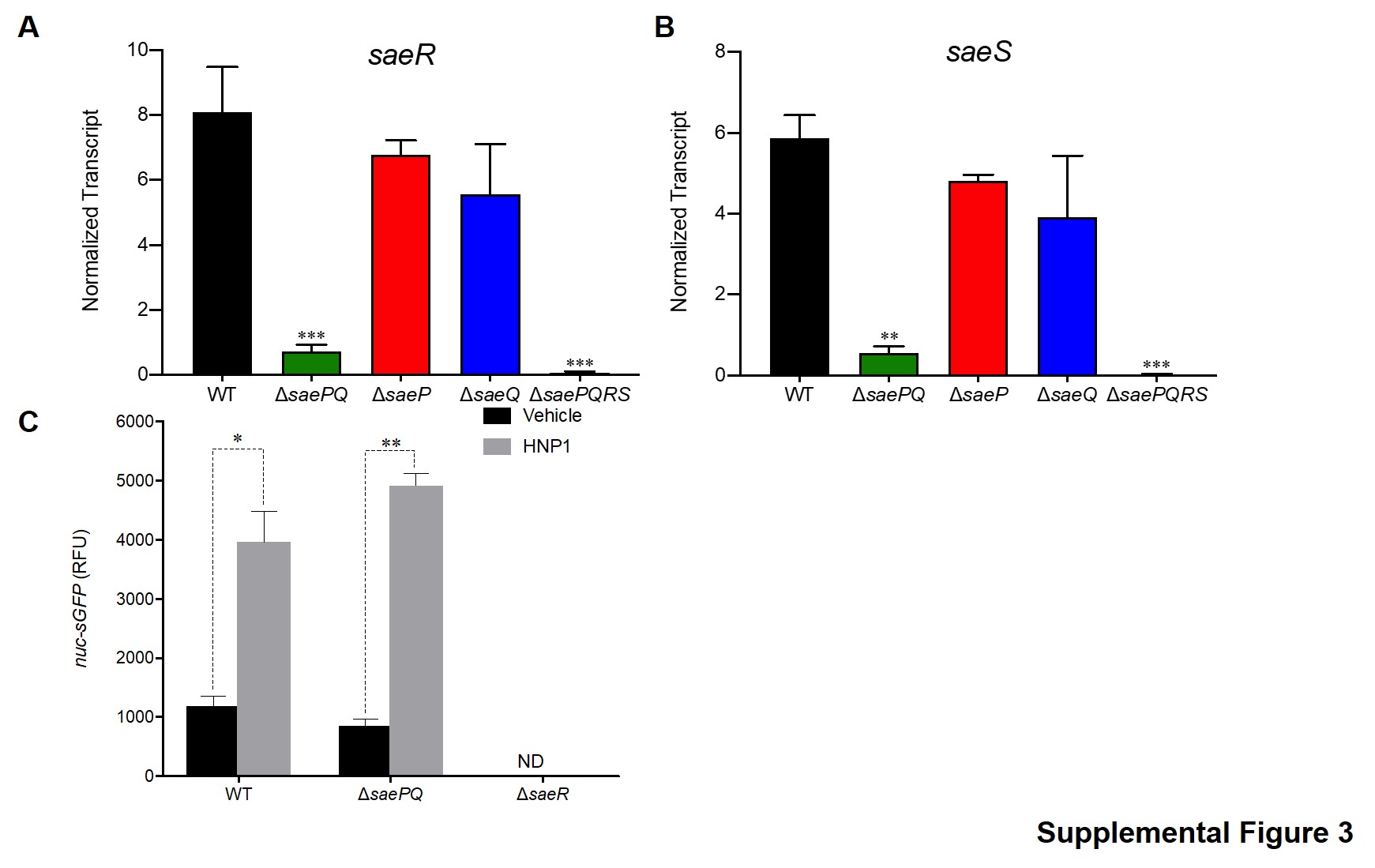

Supplement: FIGURE S3 — The ΔsaePQ mutant has reduced saeR/S transcript levels but induces Sae-dependent genes normally. (A,B) Normalized transcript abundance is shown for USA300 and the indicated mutant strains of S. aureus grown to exponential phase using SYBR green qRT-PCR as described in Materials and Methods and as described in Mlynek et al. (2018). Data indicate the mean ± SEM from three independent experiments. Statistical analysis: ANOVA (ordinary one-way), Dunnett’s multiple comparison test, asterisks indicate the level of significance compared to the USA300 (P ≤ 0.05). For USA300ΔsaePQRS, the transcript level is below the limit of detection. (C) The indicated strains carrying a nuc-gfp reporter fusion were grown to exponential phase (OD600 ∼ 0.4) in dilute Luria broth, at which time bacteria were exposed to either vehicle (water) or HNP-1 for 12 h. The data shown are the mean relative fluorescence units (RFUs; fluorescence/OD600) ±SEM for three independent experiments performed in technical triplicate; statistical significance was assessed by paired t-test (∗P ≤ 0.05, ∗∗P ≤ 0.01, ∗∗∗P ≤ 0.001). ND, not detected: signal was below the limit of detection. [file Image_3.jpeg]

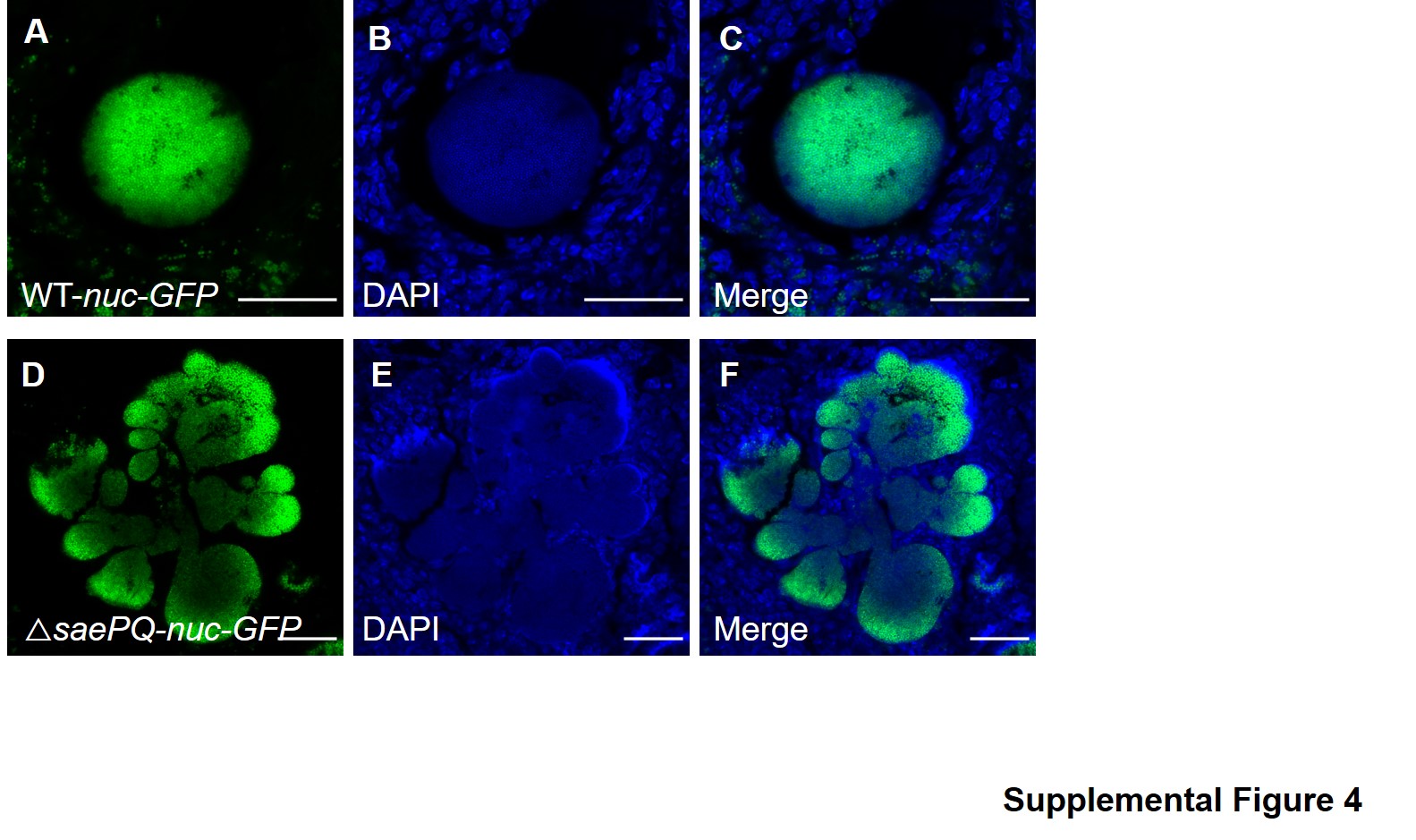

Supplement: FIGURE S4 — Deletion of saePQ influences lesion structure and nuc expression in infected kidneys. (A–F) Representative confocal micrographs of staphylococcal lesions in kidney produced by USA300 and USA300ΔsaePQ. Channels: nuc-sGFP (A,D), DAPI (B,E), and merge (C,F) (scale bar is 25 μm). The fluorescence image acquisition parameters used are as follows; excitation for DAPI (blue) and GFP (green) are 405 and 488 nm respectively; emission ranges are 419–481 nm (DAPI) and 505–551 nm (GFP). [file Image_4.jpeg]
